# Monocytic MDSC mobilization promotes tumor recurrence after liver transplantation via CXCL10/TLR4/MMP14 signaling

**DOI:** 10.1038/s41419-021-03788-4

**Published:** 2021-05-14

**Authors:** Hui Liu, Chang Chun Ling, Wai Ho Oscar Yeung, Li Pang, Jiang Liu, Jie Zhou, Wei Yi Zhang, Xiao Bing Liu, Tak Pan Kevin Ng, Xin Xiang Yang, Chung Mau Lo, Kwan Man

**Affiliations:** 1 Department of Surgery, HKU-SZH & Faculty of Medicine, The University of Hong Kong, Hong Kong, China; 2 Department of General Surgery, Affiliated Hospital of Nantong University, Nantong, 226001 China

**Keywords:** Liver cancer, Experimental models of disease

## Abstract

Tumor recurrence is the major obstacle for pushing the envelope of liver transplantation for hepatocellular carcinoma (HCC) patients. The inflammatory cascades activated by acute liver graft injury promote tumor recurrence. We aimed to explore the role and mechanism of myeloid-derived suppressor cell (MDSC) mobilization induced by liver graft injury on tumor recurrence. By analyzing 331 HCC patients who received liver transplantation, the patients with graft weight ratio (GWR, the weight of liver graft divided by the estimated standard liver weight of recipient) <60% had higher tumor recurrence than GWR ≥60% ones. MDSCs and *CXCL10/TLR4* levels were significantly increased in patients with GWR <60% or tumor recurrence. These findings were further validated in our rat orthotopic liver transplantation model. In *CXCL10*^−/−^ and *TLR4*^−/−^ mice of hepatic ischemia/reperfusion injury plus major hepatectomy (IRH) model, monocytic MDSCs, instead of granulocytic MDSCs, were significantly decreased. Importantly, *CXCL10* deficiency reduced the accumulation of TLR4^+^ monocytic MDSCs, and CXCL10 increased MDSC mobilization in the presence of TLR4. Moreover, MMP14 was identified as the key molecule bridging CXCL10/TLR4 signaling and MDSC mobilization. Knockout or inhibition of CXCL10/TLR4 signaling significantly reduced the tumor growth with decreased monocytic MDSCs and MMP14 in the mouse tumor recurrent model. Our data indicated that monocytic MDSCs were mobilized and recruited to liver graft during acute phase injury, and to promote HCC recurrence after transplantation. Targeting MDSC mobilization via CXCL10/TLR4/MMP14 signaling may represent the therapeutic potential in decreasing post-transplant liver tumor recurrence.

## Introduction

Liver transplantation offers an effective therapeutic treatment for selected patients with hepatocellular carcinoma (HCC), which is the third cause of cancer-related mortality worldwide^1,2^. The advances of living donor liver transplantation (LDLT) relieve the drastic shortage of donor pool, shorten the waiting time and decrease the waiting-list mortality^3^. However, several liver transplantation centers reported that LDLT patients experienced higher HCC recurrence in contrast with deceased donor liver transplantation (DDLT)^4,5^. The liver graft in LDLT is usually small-for-size for the recipients, which is susceptible to severer graft injury at the acute phase. The inflammatory responses resulted from liver graft injury may cause tumor cells more aggressive by triggering tumor cell adhesion, migration, and invasion^3,6^. Animal studies showed that attenuation of hepatic ischemia/reperfusion injury could effectively decrease liver cancer recurrence^7^. Nevertheless, the mechanism of small-for-size liver graft injury promoting cancer recurrence has not been well illustrated.

Chemokine (C-X-C motif) ligand 10 (CXCL10), also known as interferon-gamma inducible protein (IP-10), plays a critical role in recruiting the immune cells infiltrated into the liver in hepatic diseases^8,9^. In the development of hepatic ischemia/reperfusion injury, it has been identified that CXCL10 is required for the induction of pro-inflammatory responses^10^. Oncologically, CXCL10, with its receptor C-X-C motif chemokine receptor 3 (CXCR3), facilitates cancer cell proliferation, metastasis, and invasion^11–13^. CXCL10 also contributes to inflammatory responses or hepatocellular apoptosis through toll-like receptor 4 (TLR4), but not CXCR3^14,15^. The intestinal venous congestion caused by liver graft injury facilitated cancer recurrence by TLR4 increase in rodent models^16^. However, few studies reported the mechanism of CXCL10/TLR4 signaling regulating immune cells for promoting liver cancer recurrence.

A key cell population defined as myeloid-derived suppressor cells (MDSCs) contributes to the immunosuppression in tumors, which is critical in tumor evasion of the immune system^17^. MDSC can disrupt immune surveillance by suppressing effector T cells^18^, blocking nature killer cell cytotoxicity^19^, expanding regulatory T cells^20^, and skewing macrophages into immunosuppressive M2 phenotype^21^. The liver is a favorable site for MDSC expansion^17^. Recent studies indicated that the hypoxia environment recruited MDSCs to promote HCC^22,23^. Chemokines modulate the hepatic microenvironment and regulate critical aspects in the pathogenesis of liver cancer^24^. However, the relationship of chemokines, especially CXCL10 signals, with MDSC mobilization in HCC recurrence after transplantation has never been explored.

In this study, we aimed to explore the role and mechanism of MDSC mobilization by CXCL10 signaling at early graft injury, which might lead to tumor recurrence post-liver transplantation. We demonstrated the association among acute-phase MDSC recruitment, CXCL10/TLR4 overexpression, and tumor recurrence after small-for-size liver graft transplantation in both clinical and rat transplant studies. Furthermore, we provided new insights that CXCL10 through TLR4, instead of CXCR3, mobilized monocytic MDSCs, but not granulocytic MDSCs, to the liver for facilitating cancer recurrence using *CXCL10*^−*/*−^, *CXCR3*^−*/*−^ and *TLR4*^−*/*−^ mice. Higher TLR4 expression on monocytic MDSCs might explain why the monocytic MDSCs were mobilized. The direct role of CXCL10 on MDSC mobilization through TLR4 was verified through in vitro studies. We also discovered a motility-related molecule, matrix metallopeptidase 14 (MMP14), bridged CXCL10/TLR4 signaling and MDSC mobilization.

## Materials and methods

### Clinical cohort and biopsies

From 1995 to 2016, 331 patients with HCC who underwent liver transplantation in Queen Mary Hospital, The University of Hong Kong, were included in our analysis. Among them, 50 patients experienced HCC recurrence (recurrence) while the other 281 patients had no HCC recurrence (non-recurrence). The graft weight ratio (%) (GWR) has been defined as the weight of liver graft divided by the estimated standard liver weight of the recipient^25^. According to the GWR, the patients were assigned as large liver graft group (GWR ≥60%, *n* = 149) and small-for-size liver graft group (GWR < 60%, *n* = 180). The liver graft biopsies and blood were collected at 2 h and 7 days after portal vein reperfusion, respectively. All clinical record access and tissue sample collection were approved with signed consent forms by each donor and recipient. The procedures followed in this study was in accordance with the ethical standards of the Institutional Review Board (IRB) of The University of Hong Kong and with the Helsinki declaration of 1975, as revised in 1983. No donor organs were obtained from executed prisoners or other institutionalized persons.

### Animal models

Male Sprague Dawley (SD), Buffalo rats, and C57 BL/6 mice (6–8 weeks) were purchased from the Laboratory Animal Unit, The University of Hong Kong. The CXCL10, CXCR3, and TLR4 knockout mice (6–8 weeks) were described in previous papers with the controls cohoused^26,27^. All animals were housed in a standard animal facility at 22 ± 2 °C under controlled 12-h light/dark cycles. Both rats and mice had free access to regular chow (5053-PicoLab^®^Rodent Diet 20, Lab Diet, MO, USA) and autoclaved water. According to our preliminary experiment, five animals were chosen in each group (*N* = 5). All the animals were randomly grouping with no blinding. The animals with poor physical conditions were excluded. All animals received humane care according to the criteria outlined in *Guide for the Care and Use of Laboratory Animals* (*National Institutes Health publication 86–23, 1985 revision*). The experimental protocols were performed under the guidelines approved by the Committee on the Use of Live Animals in Teaching and Research, The University of Hong Kong

(1) *Rat orthotopic liver transplantation model*

The orthotopic liver transplantation model was established using whole graft and small-for-size graft (ratio of graft weight to recipient liver weight was about 50%) in SD rats. Blood and liver tissues were collected on Day 1, 3, 5 after transplantation to investigate MDSC mobilization and CXCL10/TLR4 levels in early-stage liver graft injury. The management of rat orthotopic liver transplantation was implemented according to the surgical protocol described previously^28,29^. To analyze the MDSC distribution, CXCL10/TLR4 expressions, and intragraft angiogenesis in recurrent tumors, a rat orthotopic liver transplantation with tumor recurrence model in Buffalo rats were used as described in our previous papers^30,31^. The tumor and non-tumor tissues of the liver graft were collected at day 14 post-transplantation.

(2) *Mouse hepatic ischemia/reperfusion plus major hepatectomy (IRH) model*

To mimic liver transplantation using a small-for-size graft, the wild type (C57 BL/6), *CXCL10*^−*/*−^, *CXCR3*^−*/*−^, and *TLR4*^−*/*−^ mice were subjected to partial ischemia/reperfusion injury plus major hepatectomy^26^. The right branch of the portal vein and hepatic arterial was clamped for 45 min, meanwhile, the left and caudate lobes were removed before reperfusion. Blood and liver tissues were collected on Day 1, 3, 5 after reperfusion.

The mouse liver tumor cells (Hepa1-6, ATCC, 2 × 10^6^/100 μl per mouse) were injected into the portal vein immediately after hepatic IRH to establish mouse liver IRH with tumor recurrence model. To investigate the role of TLR4 on MDSC mobilization in liver tumor recurrence, CLI095 (TLR4 inhibitor, 3 mg/kg, Invivogen, CA, USA) was injected intraperitoneally 2 h before the operation and every two days after the reperfusion. About 21 days after the surgical process, blood and liver tissues were collected for analysis.

### Primary MDSC isolation by magnetic bead cell sorting from mouse spleen and bone marrow

Bone marrow cells were obtained from femurs and tibias of mice. The splenic cells and bone marrow cells were flushed out using phosphate-buffered saline by syringe. The cell suspensions were filtered through 70 μm cell strainers and treated with ACK lysing buffer (Chem Cruz, Santa Cruz Biotechnology, TX, USA). After obtaining the single-cell suspension, Gr1^+^CD11b^+^ cells (MDSCs) were isolated using a mouse MDSC isolation kit (Stemcell Technologies, BC, Canada), according to the manufacturer’s instruction. The MDSC purity was evaluated by Gr1^+^CD11b^+^ (Stemcell Technologies; BD Pharmingen, CA, USA) population >90% via flow cytometry.

### Laboratory methods

Further details on Flow cytometry analysis, H&E, and immunostaining, qRT-PCR, MDSC transwell assay, RT2 profiler PCR array can be found in the Supplementary methods section.

### Statistical analysis

Categorical data were analyzed by Chi-square/Fisher’s exact test. Comparison of continuous variables was performed by Student’s *t*-test/Mann–Whitney *U* test where appropriate. Clinical survival analysis was implemented using Kaplan–Meier test. Data were presented as mean ± SEM (standard error of the mean). Degrees of statistical significance was indicated using standardized asterisk nomenclature (*p < 0.05, **p < 0.01, ***p < 0.001). All analyses were performed with SPSS18.0 (SPSS, IL, USA) and Graphpad Prism 5.0 (GraphPad Software Inc, CA, USA).

## Results

### Tumor recurrent rate was higher in HCC patients after small-for-size liver graft transplantation

**Fig. 1:**
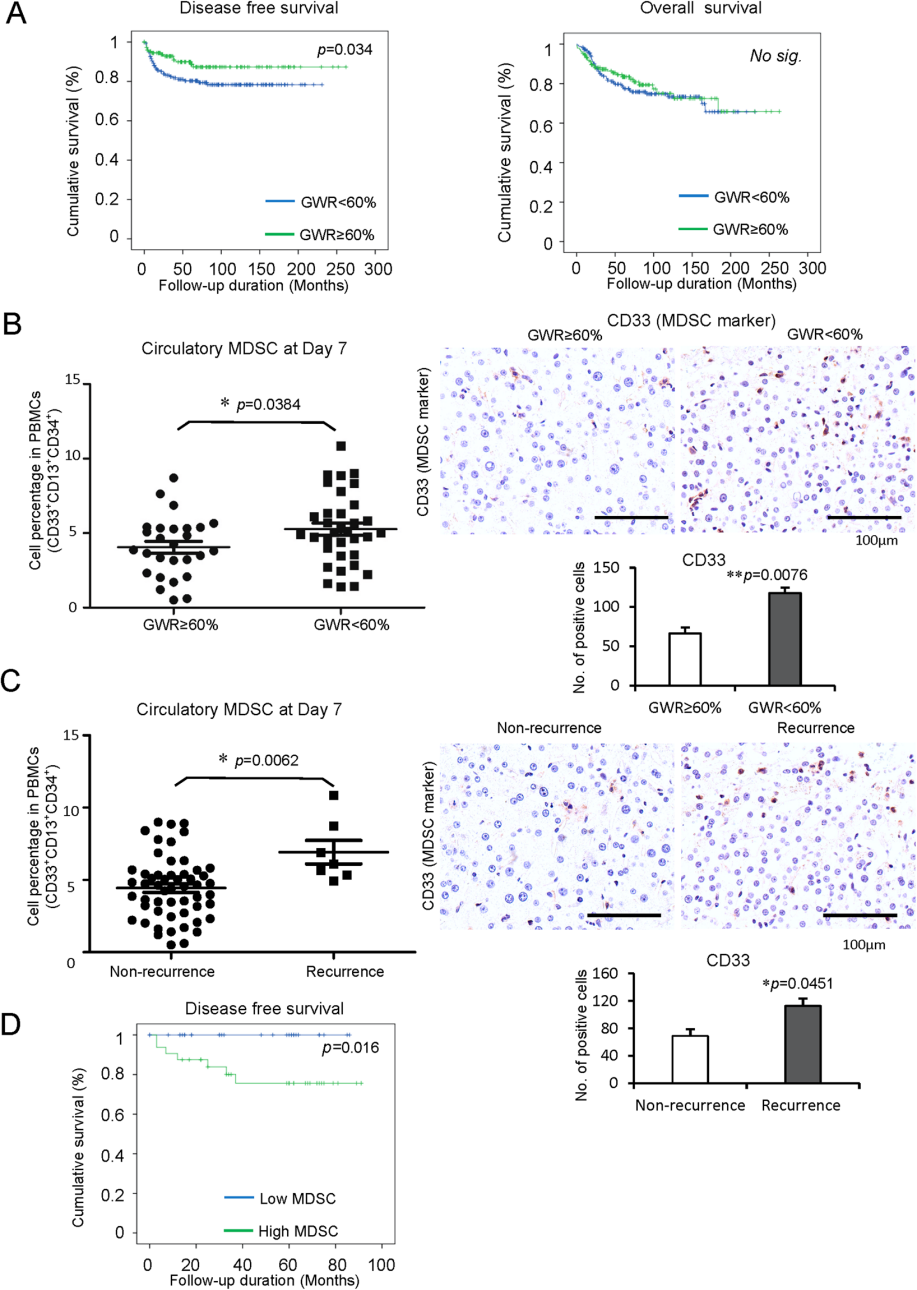
Increased MDSCs in HCC patients with small-for-size graft or tumor recurrence accompanied by poor disease-free survival post-transplantation. The circulatory and intragraft MDSCs were detected on Day 7 and hour 2 after liver transplantation, respectively. **A** The patients who received small-for-size liver grafts (GWR < 60%) had poor disease-free survival than patients with large liver grafts (GWR ≥ 60%) (*n* = 331). **B** Increased circulatory and intragraft MDSCs in patients with small-for-size grafts compared to those with large liver grafts (*n* = 61). **C** More circulatory and intragraft MDSCs in patients experienced HCC recurrence in contrast with non-recurrent patients (*n* = 61). **D** Poor disease-free survival in HCC patients with high MDSCs post-transplantation (*n* = 61). Scale bars: 100 μm. Error bars indicate standard error of mean; *p < 0.05, **p < 0.01. MDSC myeloid-derived suppressor cell, HCC hepatocellular carcinoma, GWR graft weight ratio.

Three hundred and thirty-one HCC patients who received liver transplantation were recruited in this study. HCC recurrence occurred in 50 patients post-liver transplantation. According to the analysis of clinical parameters, the recurrence was more frequent in the patients with positive HBsAg (*p* = 0.01), higher serum alpha-fetoprotein (AFP) (*p* = 0.000), more number of tumors (*p* = 0.002), the larger size of tumor largest size (*p* = 0.003), macrovascular invasion (*p* = 0.000), advanced TNM stage (*p* = 0.000), beyond Milan criteria (*p* = 0.000) and UCSF criteria (*p* = 0.000), vascular permeation (*p* = 0.000) and higher differentiation of tumor (*p* = 0.025). Importantly, tumor recurrent rate was significantly higher in the HCC patients with small-for-size graft (GWR < 60%) post-liver transplantation (*p* = 0.018) (Table 1). Consistent with the results, the survival analysis demonstrated that the patients with GWR < 60% had poor disease-free survival compared to patients with GWR ≥ 60%, while no significant difference in overall survival after liver transplantation (Fig. 1A).

**Table 1:** Comparison of baseline characteristics between non-recurrent and recurrent HCC patients after liver transplantation.

	Non-recurrence (*N* = 281)	Recurrence (*N* = 50)	*p* value
Gender (*n*)			0.095
Male	225	45	
Female	56	5	
Age (years)	55.38 ± 0.475	52.14 ± 1.146	0.006*
HBsAg (*n*)			0.01*
Negative	61	3	
Positive	220	47	
AFP (ng/ml)	477.88 ± 164.67	4882.72 ± 2509.78	0.000*
No. of tumors (*n*)^a^	1.94 ± 0.10	3.34 ± 0.43	0.002*
The largest size of tumor (cm)^a^	3.02 ± 0.08	4.15 ± 0.43	0.003*
Macrovascular invasion (*n*)^a^			0.000*
No	264	39	
Yes	7	9	
TNM staging (*n*)^a^			0.000*
I	109	9	
II	144	25	
III	14	13	
Milan criteria (*n*)^a^			0.000*
Within criteria	192	20	
Beyond criteria	81	29	
UCSF criteria (*n*)^a^			0.000*
Within criteria	222	21	
Beyond criteria	51	28	
Vascular permeation (*n*)^a^			0.000*
No	190	18	
Yes	71	29	
Tumor differentiation (*n*)^a^			0.025*
Well-differentiated	78	5	
Moderately differentiated	156	33	
Poorly differentiated	12	5	
Undifferentiated	2	0	
Graft weight to recipient ESLV (%) (*n*)^a^			0.018*
<60%	145	35	
≥60%	134	15	

*ESLV* estimated standard liver volume.

*p < 0.05.

^a^Total number less than 331 due to missing data.

### MDSCs were increased in HCC patients with small-for-size graft or tumor recurrence post-liver transplantation

More circulatory (Day 7, *p* = 0.0384) and intragraft (2 h post perfusion, *p* = 0.0076) MDSCs were found in the patients with small-for-size graft compared to large graft post-liver transplantation (Fig. 1B). Furthermore, the patients with HCC recurrence had a higher population of the circulatory (Day 7, *p* = 0.0062) and intragraft (2 h post perfusion, *p* = 0.0451) MDSCs than non-recurrent patients after transplantation (Fig. 1C). The patients with high MDSCs had poor disease-free survival (Fig. 1D). It suggested that MDSCs may play important roles in tumor recurrence after liver transplantation for HCC.

### Higher CXCL10/TLR4 levels in small-for-size grafts were associated with tumor recurrence in HCC patients post-liver transplantation

**Fig. 2:**
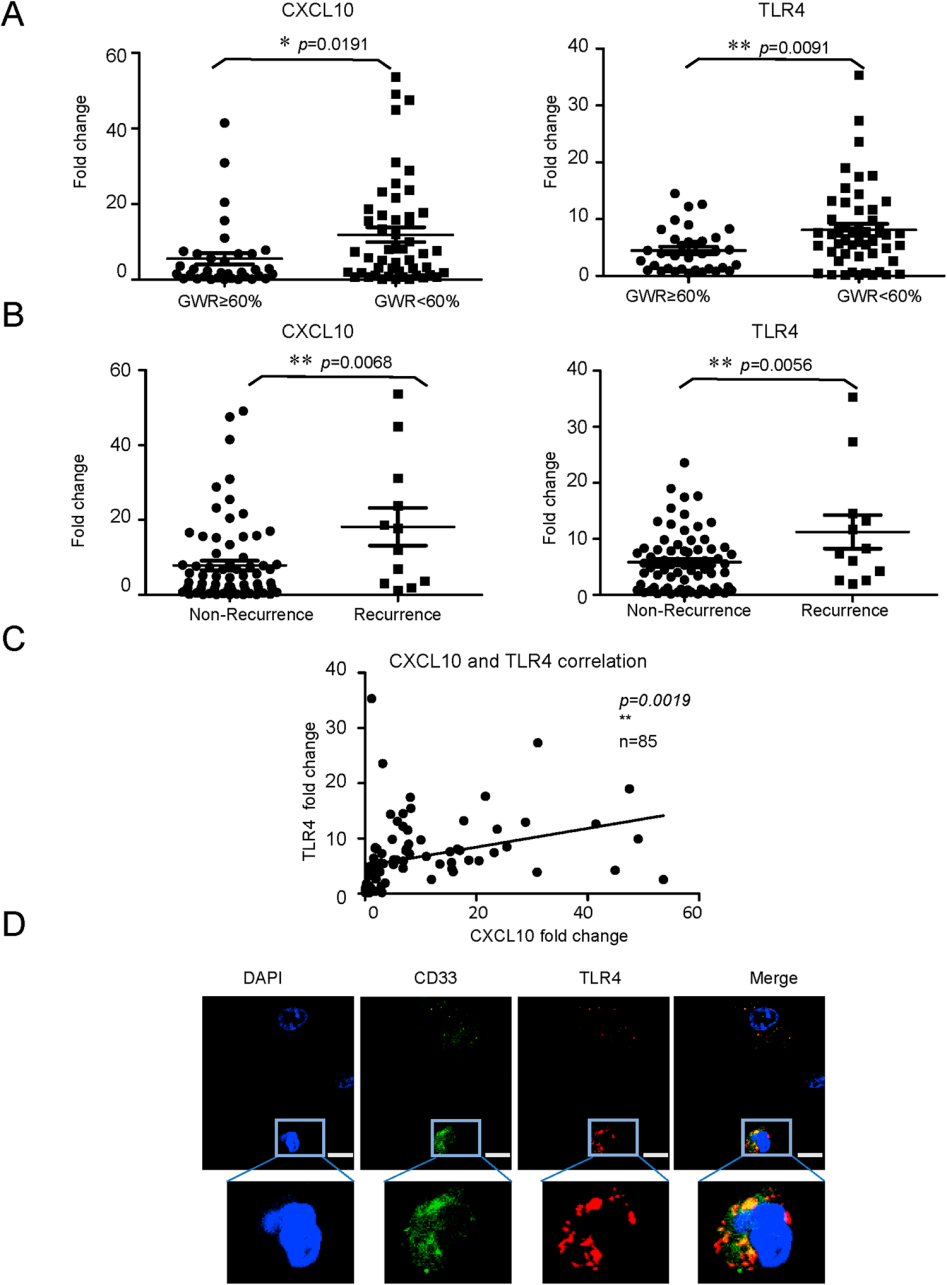
Association of higher CXCL10/TLR4 levels in small-for-size graft with tumor recurrence in HCC patients after transplantation. Intragraft CXCL10 and TLR4 expressions were examined at hour 2 after liver transplantation. **A** Enhanced expression of CXCL10 and TLR4 in patients who received small-for-size liver grafts (GWR < 60%) compared to large liver grafts (GWR ≥ 60%) (*n* = 85). **B** Higher levels of CXCL10 and TLR4 in the recipients with HCC recurrence than non-recurrent patients (*n* = 85). **C** The significant positive correlation between intragraft CXCL10 and TLR4 expression (*n* = 85). **D** Co-localization of TLR4 and MDSC (CD33 positive) in liver graft post-transplantation (a representative from three times independent experiments). Scale bars: 5 μm. Error bars indicate standard error of mean; *p < 0.05, **p < 0.01. HCC hepatocellular carcinoma, MDSC myeloid-derived suppressor cell, GWR graft weight ratio.

The intragraft levels of CXCL10 (*p* = 0.0191) and TLR4 (*p* = 0.0091) were significantly higher in patients with small-for-size grafts in contrast with large grafts at 2 h after reperfusion (Fig. 2A). Consistent with the results of MDSC analysis, the intragraft expressions of CXCL10 (*p* = 0.0068) and TLR4 (*p* = 0.0056) were also significantly up-regulated in the patients with HCC recurrence in contrast with those without HCC recurrence (Fig. 2B). Importantly, the intragraft expressions of CXCL10 and TLR4 were highly correlated (*p* = 0.0019, Fig. 2C). The co-localization of TLR4 and MDSC marker (CD33) further demonstrated that CXCL10 might play the role through TLR4 on MDSC mobilization post-transplantation (Fig. 2D).

### MDSC mobilization and CXCL10/TLR4 expressions were associated with liver tumor recurrence after rat orthotopic liver transplantation

**Fig. 3:**
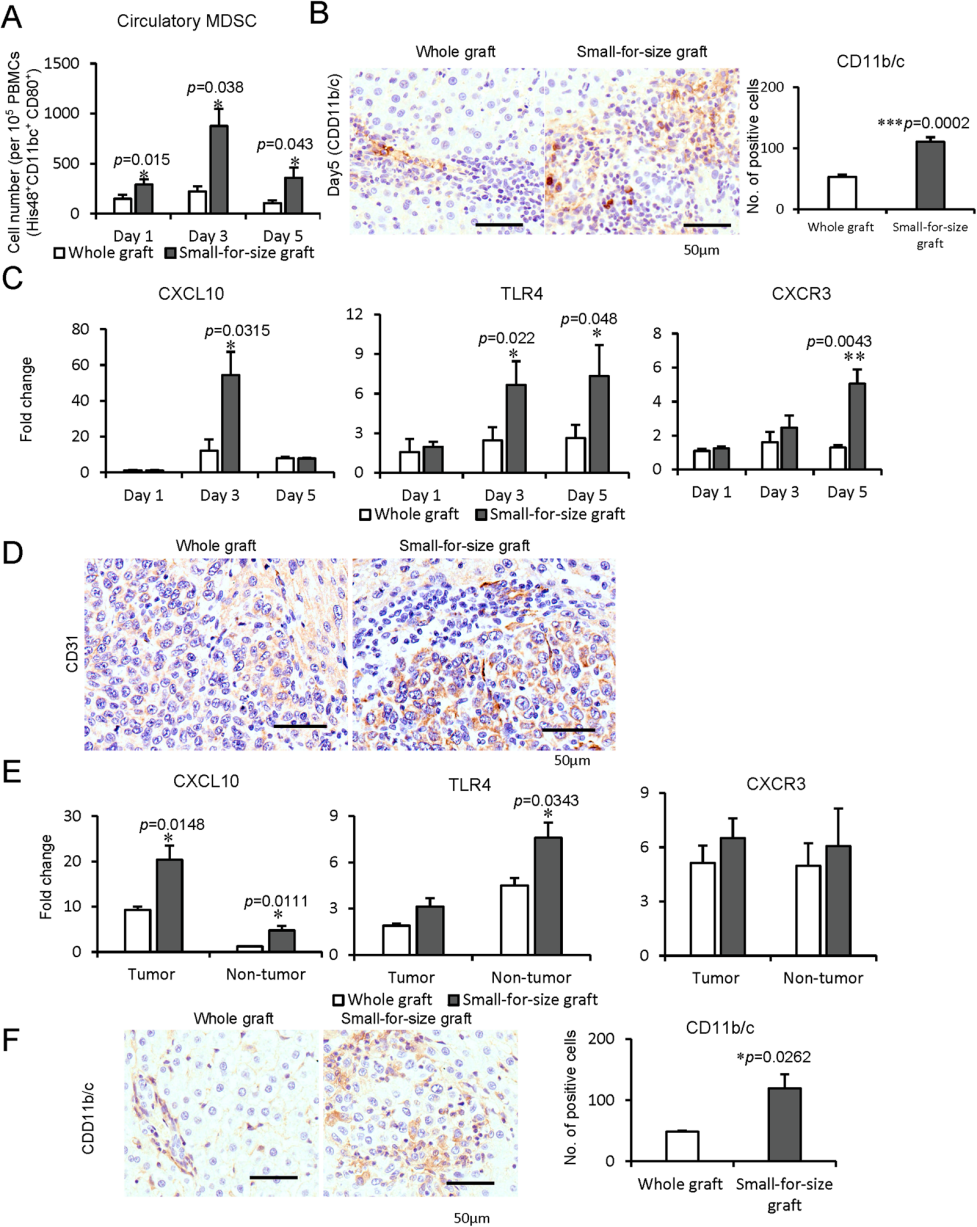
Association among MDSC mobilization, CXCL10/TLR4 expressions, and liver tumor recurrence in rat orthotopic liver transplantation models. **A**–**C** Rat orthotopic liver transplantation model. **A** More circulatory MDSCs in rats at Day 1, 3, 5 after small-for-size graft transplantation in contrast with whole graft. **B** Increased intragraft MDSCs (CD11b/c positive) in rats with small-for-size grafts at Day 5. **C** Increased intragraft mRNA expressions of CXCL10 (Day 3), TLR4 (Day 3, 5), CXCR3 (Day 5) in small-for-size liver graft. **D**–**F** Rat orthotopic liver transplantation with tumor recurrence model. **D** Higher MVD in the tumors from a small-for-size liver graft on Day 14 after transplantation. **E** Higher expressions of CXCL10 (both in tumor and non-tumor tissue) and TLR4 (in non-tumor tissue) in small-for-size liver graft with tumor recurrence. **F** More infiltrated MDSCs in small-for-size liver graft with tumor recurrence. *N* = 5/group; Scale bars: 50 μm; error bars indicate standard error of mean; *p < 0.05, **p < 0.01, ***p < 0.001. MDSC myeloid-derived suppressor cell, MVD microvessel density.

To further investigate the mobilization and recruitment of MDSCs, we established the rat orthotopic liver transplantation model with whole and small-for-size graft. The circulatory MDSCs were significantly increased in the recipient rats with small-for-size graft compared to whole graft post-transplantation (Day 1: *p* = 0.015, Day 3: *p* = 0.038, Day 5: *p* = 0.043, Fig. 3A). Consistently, the intragraft MDSCs (CD11b/c positive) were significantly increased in the rats implanted with small-for-size liver graft (*p* = 0.0002, Fig. 3B). Furthermore, the intragraft levels of CXCL10 (Day 3: *p* = 0.0315), TLR4 (Day 3: *p* = 0.022, Day 5: *p* = 0.048), and CXCR3 (Day 5: *p* = 0.0043) were significantly higher in the small-for-size liver graft (Fig. 3C).

Higher intratumoral microvessel density (MVD) (CD31 positive) was found in small-for-size graft with tumor recurrence model (Fig. 3D). This finding was consistent with our previous studies^30,31^. The levels of CXCL10 in tumor/non-tumor tissue (*p* = 0.0148, *p* = 0.0111) and TLR4 (*p* = 0.0343) in non-tumor tissue, but not CXCR3, were significantly higher in small-for-size graft than whole graft post-transplantation with tumor recurrence (Fig. 3E). Furthermore, the intragraft MDSCs (CD11b/c positive) were significantly increased in small-for-size grafts with tumor recurrence (*p* = 0.0262, Fig. 3F).

### CXCL10/TLR4 deficiency decreased mobilization and recruitment of monocytic MDSCs in the mouse IRH model

**Fig. 4:**
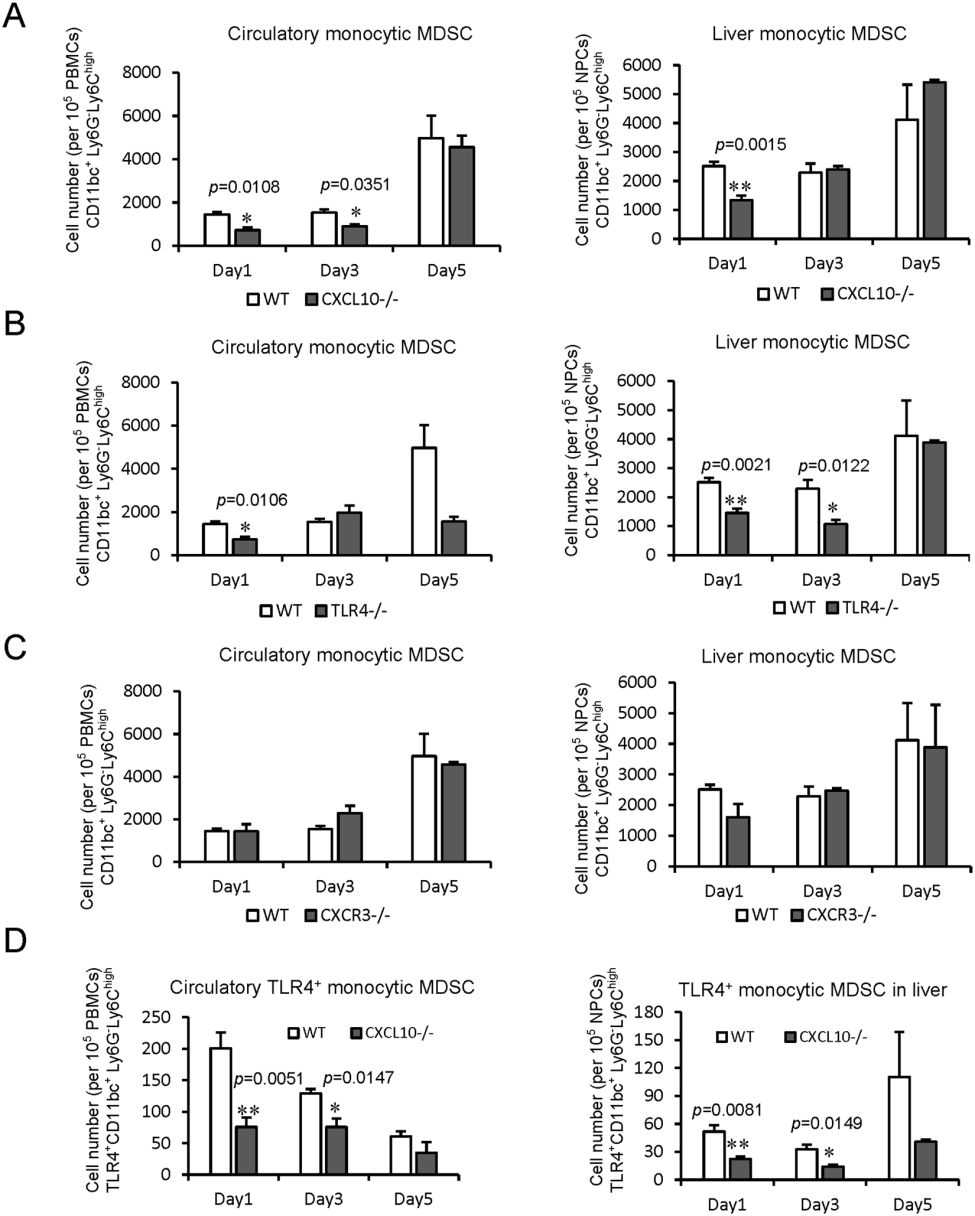
Reduced mobilization and recruitment of monocytic MDSCs by knockout of CXCL10/TLR4 in mouse IRH model. **A** Less circulatory and hepatic monocytic MDSCs in *CXCL10*^−*/*−^ mice compared to wild-type ones. **B** Significantly decreased mobilization and recruitment of monocytic MDSCs in *TLR4*^−*/*−^ mice. **C** No significant change of circulatory and hepatic monocytic MDSCs by knockout of CXCR3. **D** Reduced TLR4^+^ monocytic MDSCs in the circulation and liver of *CXCL10*^−*/*−^ mice compared to wild-type ones after IRH. *N* = 5/group; Error bars indicate Standard Error of Mean; *p < 0.05, **p < 0.01. MDSC myeloid-derived suppressor cell, IRH ischemia/reperfusion plus major hepatectomy, WT wild type.

The direct role of CXCL10 signaling on MDSC mobilization was explored in the mouse IRH model using *CXCL10*^*−/*−^, *TLR4*^−*/*−^ and *CXCR3*^−*/*−^ mice, respectively. The circulatory monocytic MDSCs were significantly reduced in *CXCL10*^−*/*−^ (Day 1: *p* = 0.0108, Day 3: *p* = 0.0351) and *TLR4*^−*/*−^ (Day 1: *p* = 0.0106) mice. Furthermore, the hepatic monocytic MDSCs were obviously decreased in *CXCL10*^−*/*−^ (Day 1: *p* = 0.0015) and *TLR4*^*−/−*^ (Day 1: *p* = 0.0021, Day 3: *p* = 0.0122) mice in contrast with wild type ones after IRH (Fig. 4A, B). Interestingly, knockout of CXCL10 and TLR4 had no effects on the mobilization and recruitment of granulocytic MDSCs (Supplementary Fig. 1A, B). Furthermore, neither the circulatory nor hepatic monocytic/granulocytic MDSCs were significantly changed in *CXCR3*^−*/*−^ mice (Fig. 4C, Supplementary Fig. 1C). Furthermore, the circulatory (Day 1: *p* = 0.0051, Day 3: *p* = 0.0147) and hepatic (Day 1: *p* = 0.0081, Day 3: *p* = 0.0149) TLR4^+^ monocytic MDSCs were both down-regulated in *CXCL10*^−*/*−^ mice compared to wild type ones (Fig. 4D). These results demonstrated that up-regulation of CXCL10, mainly through TLR4 signaling rather than CXCR3, mobilized and recruited monocytic MDSCs instead of granulocytic MDSCs to the liver to promote tumor recurrence.

### CXCL10/TLR4 regulated MDSC mobilization through MMP14

**Fig. 5:**
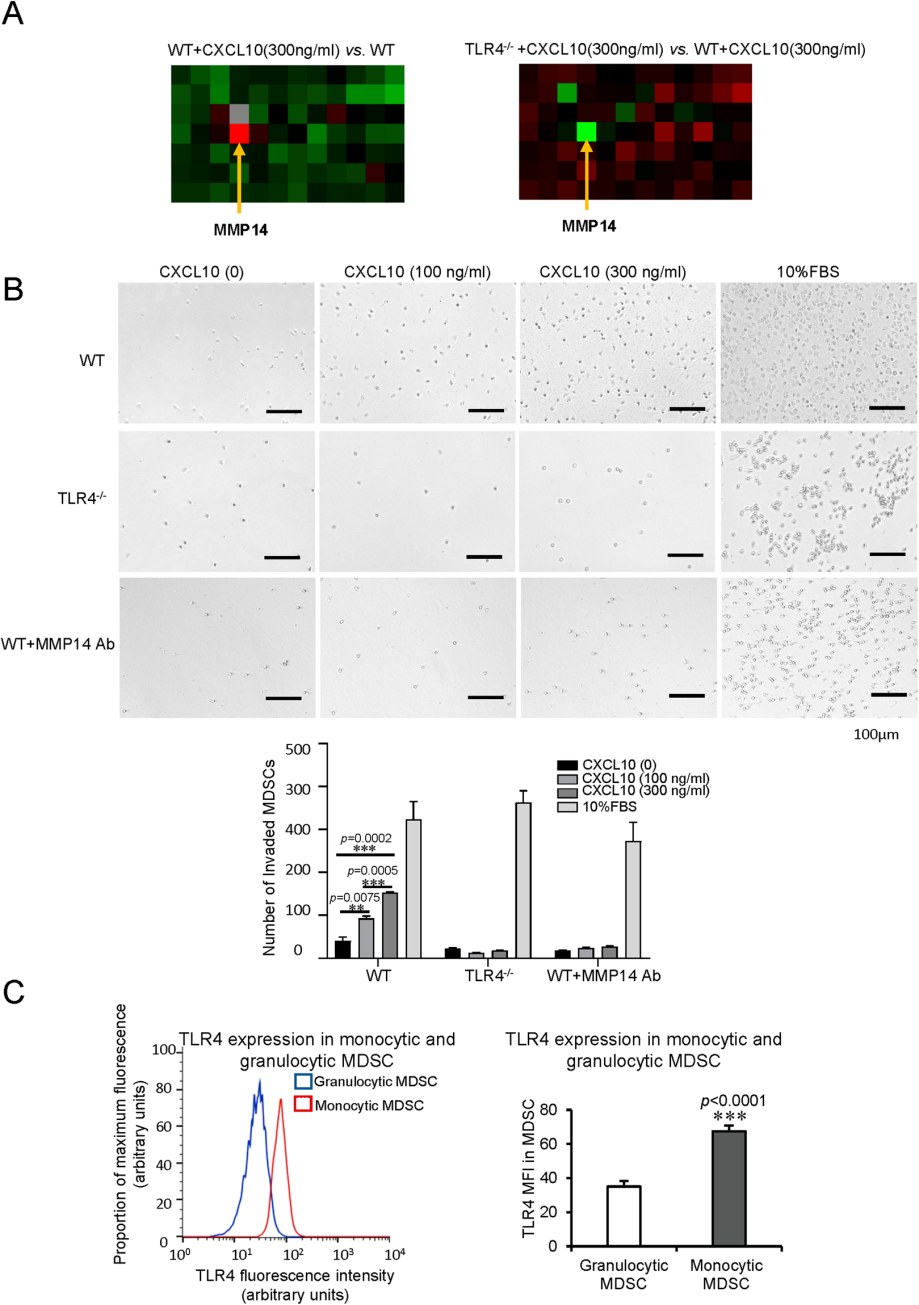
MDSC mobilization regulated by CXCL10/TLR4 through MMP14 and higher levels of TLR4 on monocytic MDSCs. **A** MMP14, as the most up-regulated gene, in wild type MDSCs with CXCL10 addition compared to those without treatment; MMP14, as the most down-regulated gene, in *TLR4*^−/−^ MDSCs compared to wild type ones with the same CXCL10 treatment by the screen of mouse motility genes. **B** More wild type MDSCs transferred to the bottom well in a CXCL10 dosage-dependent manner, while no significantly changed numbers of *TLR4*^−/−^ MDSCs and MMP14 Ab-blocking wild type MDSCs (30 μg/ml) in the bottom well with/without CXCL10 addition. **C** Augmentation of TLR4 on monocytic MDSCs in contrast with granulocytic MDSCs in mouse IRH model (*N* = 6/group). Scale bars: 100 μm. Error bars indicate standard error of mean; *p < 0.05, **p < 0.01, ***p < 0.001. MDSC myeloid-derived suppressor cell, MMP14 matrix metallopeptidase 14, Ab antibody, IRH ischemia/reperfusion plus major hepatectomy, WT wild type.

To further study the direct recruiting role of CXCL10/TLR4 on MDSCs, we isolated primary MDSCs from wild-type and *TLR4*^−/−^ mice. The purity of isolated cells was verified by flow cytometry (Supplementary Fig. 2A, B). RT2 profiler PCR array of mouse motility genes was performed from wild type and *TLR4*^−/−^ MDSCs with/without CXCL10 (300 ng/ml) treatment. The alteration of motility genes and change folds were shown in Supplementary Fig. 3A, B. Among the up-regulated genes in MDSCs with CXCL10 administration (wild type + CXCL10 vs. wild type MDSCs), MMP14 was increased most with 4.53 folds. It indicated that CXCL10 up-regulated MMP14 to increase the mobilization of MDSCs. Interestingly, among the down-regulated genes in *TLR4*^*−/−*^ MDSCs, MMP14 was also decreased most with −16.62 folds compared to wild-type ones with CXCL10 treatment (Fig. 5A, Supplementary Fig. 3B). It demonstrated that MMP14 promoting MDSC mobilization was TLR4 dependent.

In the transwell assay, recombinant CXCL10 obviously recruited more wild type MDSCs to the bottom wells in a dosage-dependent manner (100 ng/ml vs. no CXCL10: *p* = 0.0075, 300 ng/ml vs. no CXCL10: *p* = 0.0002, 300 ng/ml vs. 100 ng/ml CXCL10: *p* = 0.0005). Different from the wild type MDSCs, there was no significant change of migration for *TLR4*^−/−^ MDSCs and MMP14 antibody blocking wild type MDSCs (30 μg/ml) with or without recombinant CXCL10 addition (Fig. 5B). The result of CXCR3^−/−^ MDSCs was similar to the wild-type ones. It suggested that CXCR3 deficiency or not did not influence the MDSC migration induced by CXCL10 (Supplementary Fig. 4A). Taken together, CXCL10/TLR4 mobilized MDSCs through MMP14.

The reason why more monocytic MDSCs, but not granulocytic MDSCs, recruited to the liver after reperfusion was further explored. The TLR4 expression on monocytic MDSCs was found significantly higher compared to granulocytic MDSCs (*p* < 0.0001, Fig. 5C, Supplementary Fig. 4B) in mice. These findings indicated that more monocytic MDSCs were mobilized to the liver because of higher TLR4 expression, which was the receptor of CXCL10.

### Cancer recurrence was inhibited with reduced monocytic MDSCs by knockout or inhibition of CXCL10/TLR4 in the mouse hepatic IRH with tumor recurrence model

(1) *In CXCL10*^−/−^ *and TLR4*^−/−^ *mice*

**Fig. 6:**
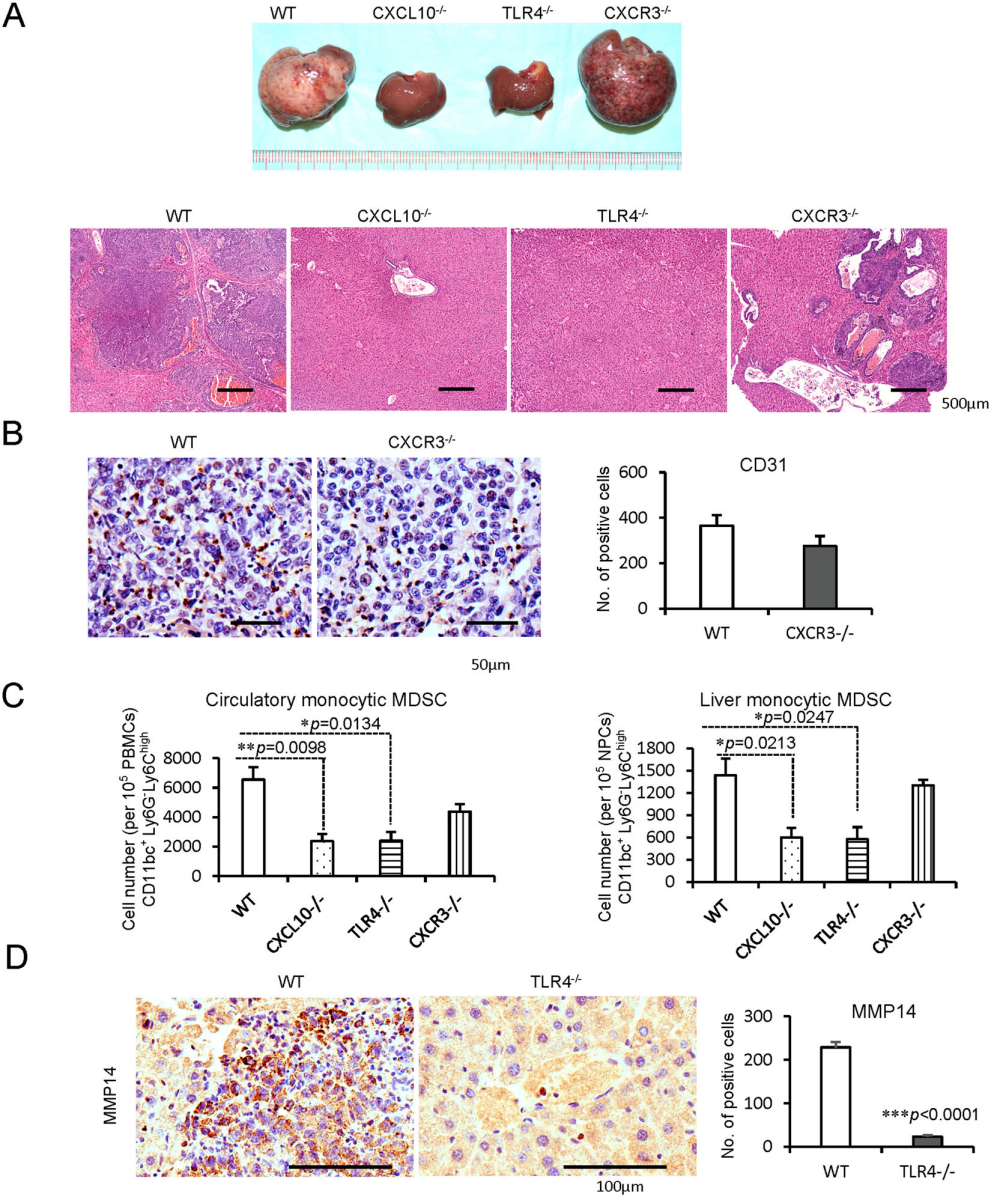
Inhibited cancer recurrence with reduced monocytic MDSCs and MMP14 in *CXCL10*^−/−^ or *TLR4*^−/−^ mice of IRH with tumor recurrence model. **A** Inhibited tumor development by knockout of CXCL10 or TLR4; scale bars: 500 μm. **B** No significant change of infiltrated CD31 positive cells in tumor tissue between wild type and *CXCR3*^−/−^ mice; Scale bars: 50 μm. **C** Less circulatory and hepatic monocytic MDSCs in *CXCL10*^−/−^ or *TLR4*^−/−^ mice. **D** Significant decrease of infiltrated MMP14 in *TLR4*^−/−^ mice in contrast with wild-type ones; scale bars: 100 μm. *N* = 5/group; error bars indicate standard error of mean; *p < 0.05, **p < 0.01, ***p < 0.001. MDSC myeloid-derived suppressor cell, MMP14 matrix metallopeptidase 14, IRH ischemia/reperfusion plus major hepatectomy, WT wild type.

To explore the direct role of CXCL10/TLR4 signaling on MDSC mobilization and late tumor recurrence, mouse hepatic IRH with tumor recurrence model was applied. Compared to wild-type mice, knockout of CXCL10 or TLR4 significantly inhibited tumor development. However, CXCR3 deficiency could not obviously reduce the tumor burden with no significant change of CD31 positive cells in tumor tissue (Fig. 6A, B). Simultaneously, the circulatory and hepatic monocytic MDSCs were significantly decreased in *CXCL10*^−/−^ (circulatory: *p* = 0.0098; hepatic: *p* = 0.0213) and *TLR4*^−/−^ (circulatory: *p* = 0.0134; hepatic: *p* = 0.0247) mice of IRH with tumor recurrence model (Fig. 6C). Consistent with our in vitro studies, MMP14 expression was also decreased in *TLR4*^−/−^ mice (*p* < 0.0001, Fig. 6D). Although the circulatory granulocytic MDSCs were decreased by knockout of CXCL10 or TLR4, the population of hepatic granulocytic MDSCs was not changed (Supplementary Fig. 5A).

(2) *TLR4 inhibition*

**Fig. 7:**
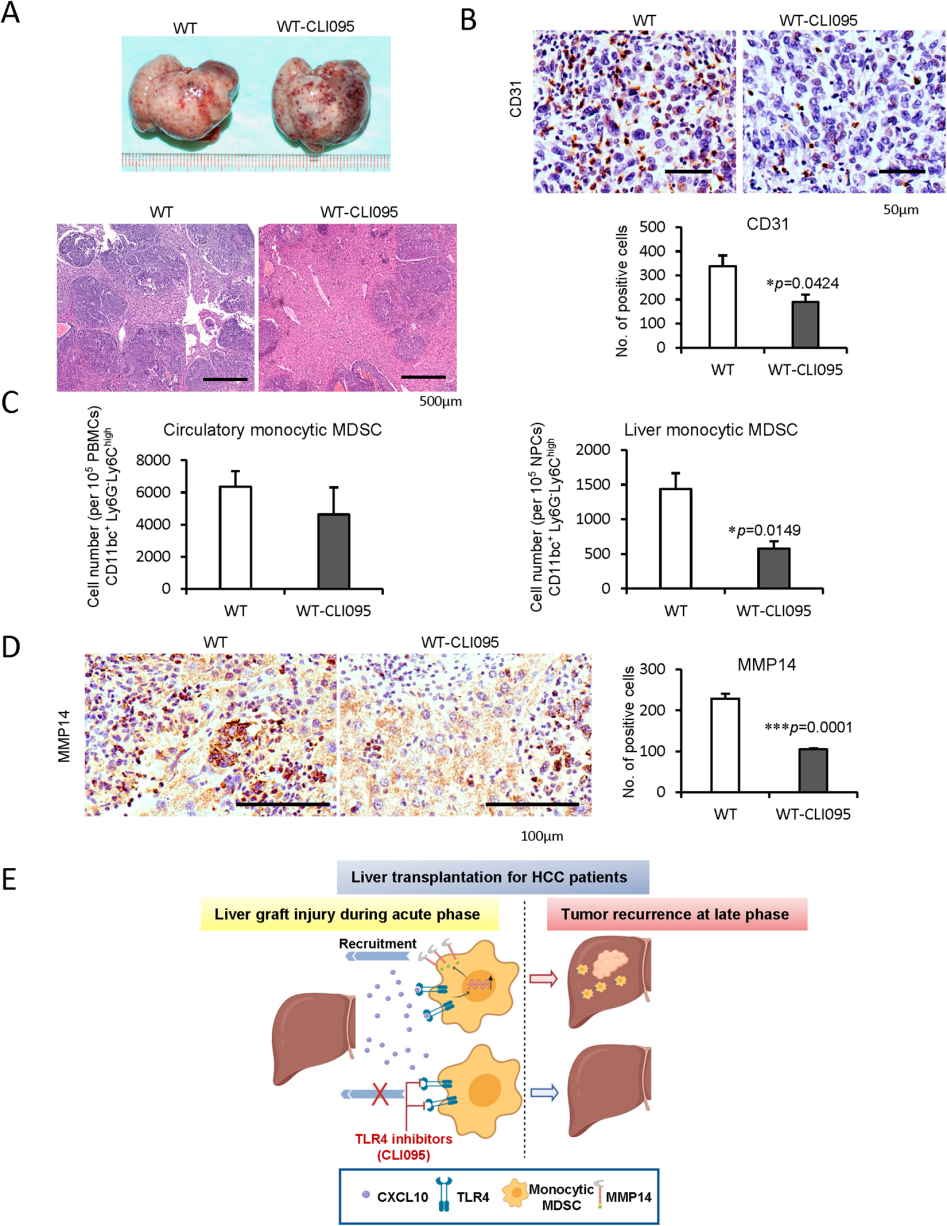
Decrease of cancer recurrence, monocytic MDSCs, and MMP14 by TLR4 inhibition in mouse liver IRH with tumor recurrence model. **A** Reduced tumor progression by the treatment of TLR4 inhibitors (CLI095) compared to no treatment wild type mice; scale bars: 500 μm. **B** The obvious decline of infiltrated CD31 positive cells in tumor tissue by TLR4 inhibition; scale bars: 50 μm. **C** Decreased liver monocytic MDSCs by TLR4 inhibition. **D** Less infiltrated MMP14 in the mice with the treatment of TLR4 inhibitors; scale bars: 100 μm. **E** Research summary: monocytic MDSCs were mobilized and recruited to liver graft through CXCL10/TLR4/MMP14 signaling during acute phase injury, and to promote HCC recurrence after transplantation. *N* = 5/group; error bars indicate standard error of mean; *p < 0.05, **p < 0.01, ***p < 0.001. MDSC myeloid-derived suppressor cell, MMP14 matrix metallopeptidase 14, IRH ischemia/reperfusion plus major hepatectomy, WT wild type.

TLR4 inhibitor (CLI095) was used to explore its therapeutic effects in tumor recurrence post IRH. Wild type mice with CLI095 treatment had a smaller tumor burden with less CD31 expression (MVD) in contrast with non-treated mice (Fig. 7A, B). Consistently, hepatic monocytic MDSCs were significantly reduced by CLI095 treatment compared to the non-treatment group (*p* = 0.0149) while no significant change for the circulatory monocytic MDSCs (Fig. 7C). Hepatic MMP14 level was also reduced in the CLI095 treatment group (*p* = 0.0001, Fig. 7D). The circulatory and hepatic granulocytic MDSCs were not obviously altered between the two groups (Supplementary Fig. 5B).

## Discussion

Tumor recurrence is a critical issue that affects the outcomes of liver transplantation for HCC patients. In this study, our clinical analysis of 331 HCC recipients indicated that the patients implanted with small-for-size graft had higher HCC recurrence and poor disease-free survival. These results were consistent with the previous clinical experience from both Western and Eastern cohorts^4,5,32^. Our previous animal studies also demonstrated the significance of acute-phase small-for-size graft injury on tumor growth and invasiveness after liver transplantation^30^. However, the mechanism of small-for-size liver graft injury leading to HCC recurrence is still largely unknown.

We first identified that MDSCs recruited by CXCL10/TLR4 during acute phase inflammation played a critical role in tumor recurrence after liver transplantation through a series of clinical analyses, animal models, and in vitro functional experiments. In addition to the immunosuppressive properties, MDSCs also possess protumorigenic functions such as angiogenesis, chemoresistance, and metastasis^33–35^. According to our results, more MDSCs were accumulated in the recipients with small-for-size grafts during acute injury. The early recruitment of MDSCs into the liver graft may provide a favorable environment for tumor recurrence. In the late phase, the recipients with small-for-size graft had more tumor recurrence with increased MDSCs compared to the whole graft. These findings indicated that MDSCs trafficking to the liver graft might be detained and proliferated, and promote the tumor recurrence. Recently, MDSCs were proposed to be applied for attenuation of allograft rejection based on their immunosuppressive function^36^. Our studies may raise the caution for using MDSCs in transplantation for cancer patients due to their role in tumor recurrence. The balance between decreasing allograft rejection and avoiding cancer recurrence for HCC recipients should be considered.

The recruitment of MDSCs to liver graft by CXCL10 is through its receptor. Intriguingly, only knockout of TLR4, instead of CXCR3, could obviously decrease the MDSC mobilization at the early stage and reduce tumor progression at the late phase. In addition, the role of CXCL10 mobilizing MDSCs via TLR4 was further validated by the high correlation of intragraft CXCL10 and TLR4, co-localization of TLR4 and MDSC marker in patients, the decrease of TLR4^+^ MDSCs in *CXCL10*^−/−^ mice and our in vitro studies. TLR4, as the pathogen recognition receptor located on the cell membrane, is responsible for the liver ischemia/reperfusion injury after transplantation^10^. In our mouse tumor recurrent model, TLR4 inhibitors could effectively reduce hepatic MDSCs and inhibit tumor growth. Therefore, targeting TLR4, could not only protect the liver graft from ischemia/reperfusion injury but also reduce MDSC mobilization to decrease tumor recurrence after transplantation.

MMP14, a component of MMP family^37^, was identified as the most up-regulated motility gene by CXCL10 addition, while the most down-regulated one in *TLR4*^−/−^ MDSCs. These findings indicated that MMP14 was the most critical molecule in MDSC migration by CXCL10/TLR4 signaling. Overexpressed MMP14 has been reported in tumor-residing MDSCs, which facilitate tumor cell invasion and metastasis of mammary carcinoma^35^. Nevertheless, the regulatory role of MMP14 on MDSCs’ motility was not clear. Our study demonstrated that the mobilization of MDSCs was mediated by MMP14, which was regulated by CXCL10/TLR4. It provided a new mechanism of MDSC motility. Thus, the overexpression of MMP14 not only facilitated tumor cell invasion but also mobilized MDSCs to the liver graft promoting HCC recurrence after transplantation. Our studies further certified that deficiency of CXCL10/TLR4 or TLR4 inhibitors could effectively reduce the monocytic MDSC recruitment with decreased MMP14, leading to the smaller tumor occupation.

Monocytic MDSCs, rather than granulocytic MDSCs, were found to be mobilized by CXCL10/TLR4 after hepatic ischemia-reperfusion. This might be explained by the higher expression of TLR4 on monocytic MDSCs in contrast to granulocytic ones. Consistently, there were more monocytic MDSCs accumulated in the mice with larger tumor volume. Monocytic and granulocytic MDSCs are the main subsets of MDSCs. Unlike terminally differentiated granulocytic MDSCs, monocytic MDSCs can differentiate into dendritic cells and macrophages^18^. The monocytic MDSCs could be one of the major sources for tumor-associated macrophages (M2 macrophages) with immunosuppressive properties. M2 macrophages have been reported to promote HCC invasiveness and recurrence in our previous study^38^. In addition, the monocytic MDSCs increase NO through STAT1/inducible nitric oxide synthase signaling. NO can suppress T-cell functions through various mechanisms to facilitate tumor progression^18^. Although the direct role of MDSCs on HCC invasiveness was not focused on in the current study, we did provide evidence that the acute phase inflammation of graft injury might initiate monocytic MDSC accumulation, which altered the liver microenvironment favoring tumor recurrence in the scenario of liver transplantation.

Taken together, the monocytic MDSCs were mobilized and recruited to the liver graft through CXCL10/TLR4/MMP14 signaling during acute phase injury, leading to HCC recurrence post-transplantation through our integrated study with clinical analyses, animal models, and in vitro experiments. Targeting CXCL10/TLR4/MMP14 to inhibit MDSC mobilization will be critical in the devising of novel therapeutic strategies against HCC recurrence after transplantation.

## Supplementary information

Supplementary information-clean

## References

[1] Villanueva A, Hernandez-Gea V, Llovet JM (2013). Medical therapies for hepatocellular carcinoma: a critical view of the evidence. Nat. Rev. Gastroenterol. Hepatol..

[2] Bray F (2018). Global cancer statistics 2018: GLOBOCAN estimates of incidence and mortality worldwide for 36 cancers in 185 countries. CA Cancer J. Clin..

[3] Man K (2017). Recurrent malignancy:are we pushing the envelope?. Liver Transpl.

[4] Fisher R (2007). Hepatocellular carcinoma recurrence and death following living and deceased donor liver transplantation. Am. J. Transpl..

[5] Kulik L (2012). Outcomes of living and deceased donor liver transplant recipients with hepatocellular carcinoma: results of the A2ALL cohort. Am. J. Transpl..

[6] Van Der Bilt JD (2005). Ischemia/reperfusion accelerates the outgrowth of hepatic micrometastases in a highly standardized murine model. Hepatology.

[7] Oldani G (2014). Pre-retrieval reperfusion decreases cancer recurrence after rat ischemic liver graft transplantation. J. Hepatol..

[8] Brownell J, Polyak SJ (2013). Molecular pathways: hepatitis C virus, CXCL10, and the inflammatory road to liver cancer. Clin. Cancer Res..

[9] Hintermann E, Bayer M, Pfeilschifter JM, Luster AD, Christen U (2010). CXCL10 promotes liver fibrosis by prevention of NK cell mediated hepatic stellate cell inactivation. J. Autoimmun..

[10] Zhai Y, Petrowsky H, Hong JC, Busuttil RW, Kupiec-Weglinski JW (2013). Ischaemia–reperfusion injury in liver transplantation—from bench to bedside. Nat. Rev. Gastroenterol. Hepatol..

[11] Datta D (2006). Ras-induced modulation of CXCL10 and its receptor splice variant CXCR3-B in MDA-MB-435 and MCF-7 cells: relevance for the development of human breast cancer. Cancer Res..

[12] Zipin-Roitman A (2007). CXCL10 promotes invasion-related properties in human colorectal carcinoma cells. Cancer Res..

[13] Wightman S (2015). Oncogenic CXCL10 signalling drives metastasis development and poor clinical outcome. Br. J. Cancer.

[14] Schulthess FT (2009). CXCL10 impairs β cell function and viability in diabetes through TLR4 signaling. Cell Metab..

[15] Sahin H (2013). Proapoptotic effects of the chemokine, CXCL 10 are mediated by the noncognate receptor TLR4 in hepatocytes. Hepatology.

[16] Orci LA (2018). Effects of the gut–liver axis on ischaemia-mediated hepatocellular carcinoma recurrence in the mouse liver. J. Hepatol..

[17] Ilkovitch D, Lopez DM (2009). The liver is a site for tumor-induced myeloid-derived suppressor cell accumulation and immunosuppression. Cancer Res..

[18] Gabrilovich DI, Nagaraj S (2009). Myeloid-derived suppressor cells as regulators of the immune system. Nat. Rev. Immunol..

[19] Liu C (2007). Expansion of spleen myeloid suppressor cells represses NK cell cytotoxicity in tumor-bearing host. Blood.

[20] Huang B (2006). Gr-1+ CD115+ immature myeloid suppressor cells mediate the development of tumor-induced T regulatory cells and T-cell anergy in tumor-bearing host. Cancer Res..

[21] Sinha P, Clements VK, Bunt SK, Albelda SM, Ostrand-Rosenberg S (2007). Cross-talk between myeloid-derived suppressor cells and macrophages subverts tumor immunity toward a type 2 response. J. Immunol..

[22] Chiu DK-C (2017). Hypoxia inducible factor HIF-1 promotes myeloid-derived suppressor cells accumulation through ENTPD2/CD39L1 in hepatocellular carcinoma. Nat. Commun..

[23] Chiu DKC (2016). Hypoxia induces myeloid-derived suppressor cell recruitment to hepatocellular carcinoma through chemokine (C-C motif) ligand 26. Hepatology.

[24] Ehling J, Tacke F (2016). Role of chemokine pathways in hepatobiliary cancer. Cancer Lett..

[25] Man K (2003). Graft injury in relation to graft size in right lobe live donor liver transplantation: a study of hepatic sinusoidal injury in correlation with portal hemodynamics and intragraft gene expression. Ann. Surg..

[26] Ling C-C (2014). Post-transplant endothelial progenitor cell mobilization via CXCL10/CXCR3 signaling promotes liver tumor growth. J. Hepatol..

[27] Ye D (2012). Toll-like receptor-4 mediates obesity-induced non-alcoholic steatohepatitis through activation of X-box binding protein-1 in mice. Gut.

[28] Man K (2001). Liver transplantation in rats using small-for-size grafts: a study of hemodynamic and morphological changes. Arch. Surg..

[29] Cheng Q (2010). Distinct mechanism of small-for-size fatty liver graft injury-wnt4 signaling activates hepatic stellate cells. Am. J. Transpl..

[30] Man K (2008). The significance of acute phase small-for-size graft injury on tumor growth and invasiveness after liver transplantation. Ann. Surg..

[31] Man K (2010). Molecular signature linked to acute phase injury and tumor invasiveness in small-for-size liver grafts. Ann. Surg..

[32] Lo C (2007). Living donor versus deceased donor liver transplantation for early irresectable hepatocellular carcinoma. Br. J. Surg..

[33] Yang L (2004). Expansion of myeloid immune suppressor Gr+ CD11b+ cells in tumor-bearing host directly promotes tumor angiogenesis. Cancer Cell.

[34] Bruchard M (2013). Chemotherapy-triggered cathepsin B release in myeloid-derived suppressor cells activates the Nlrp3 inflammasome and promotes tumor growth. Nat. Med..

[35] Yang L (2008). Abrogation of TGFβ signaling in mammary carcinomas recruits Gr-1+ CD11b+ myeloid cells that promote metastasis. Cancer Cell.

[36] Nakamura T, Ushigome H (2018). Myeloid-derived suppressor cells as a regulator of immunity in organ transplantation. Int J. Mol. Sci..

[37] Lu H (2014). KLF8 and FAK cooperatively enrich the active MMP14 on the cell surface required for the metastatic progression of breast cancer. Oncogene.

[38] Yeung OW (2015). Alternatively activated (M2) macrophages promote tumour growth and invasiveness in hepatocellular carcinoma. J. Hepatol..

