# Supplementary material for: Monocytic MDSC mobilization promotes tumor recurrence after liver transplantation via CXCL10/TLR4/MMP14 signaling
Source: Cell Death Dis. 2021 May 14;12(5):489. doi: 10.1038/s41419-021-03788-4 (PMC8121858; doi:10.1038/s41419-021-03788-4)
Supplement: Supplementary file 1 — Supplementary information-clean [file 41419_2021_3788_MOESM1_ESM.pdf]

Supplementary information

Supplementary information includes Supplementary figures, Supplementary materials and methods, Supplementary figure legends and Supplementary tables.

Supplementary Fig. 1

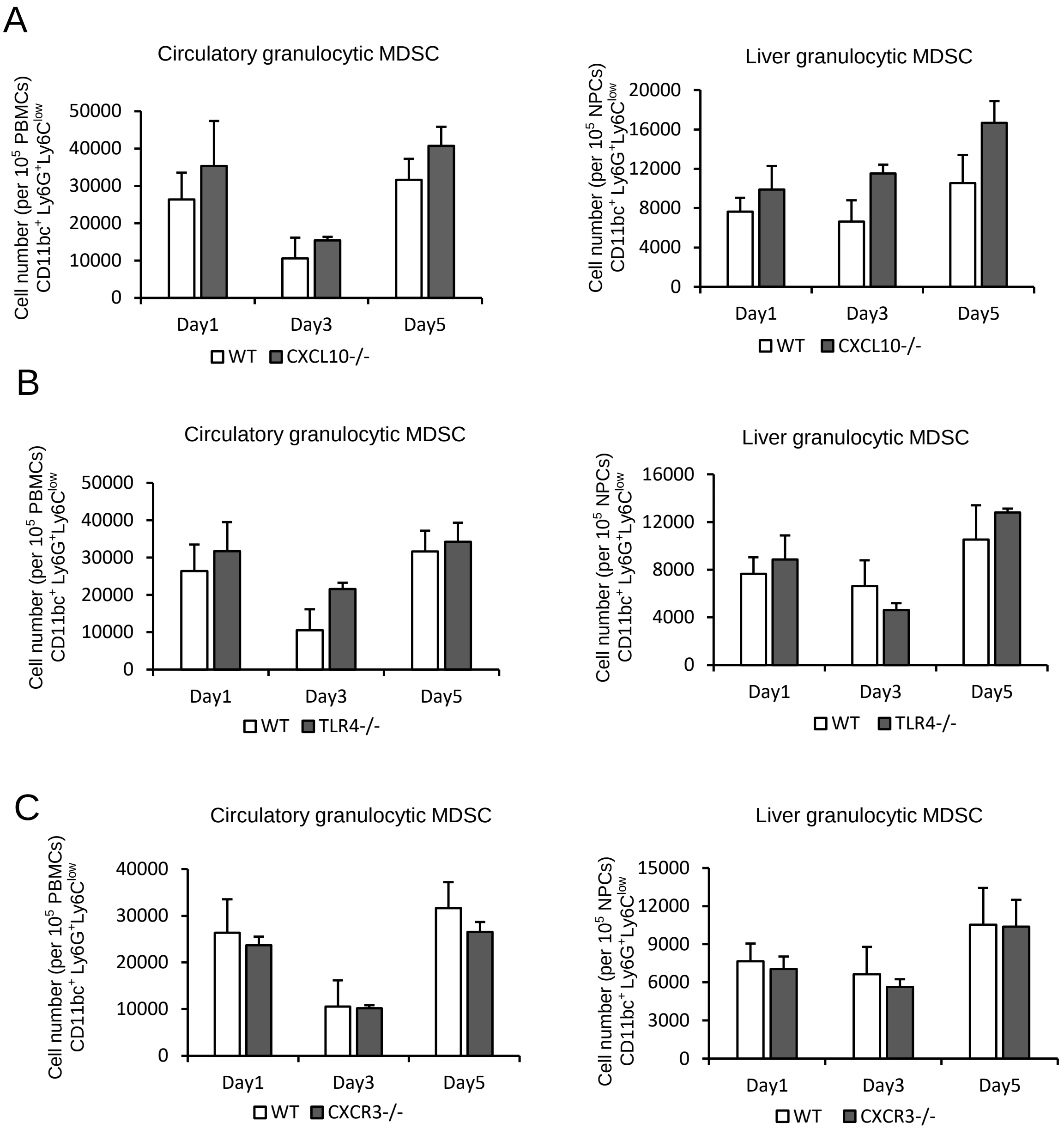

Supplementary Fig. 2

A

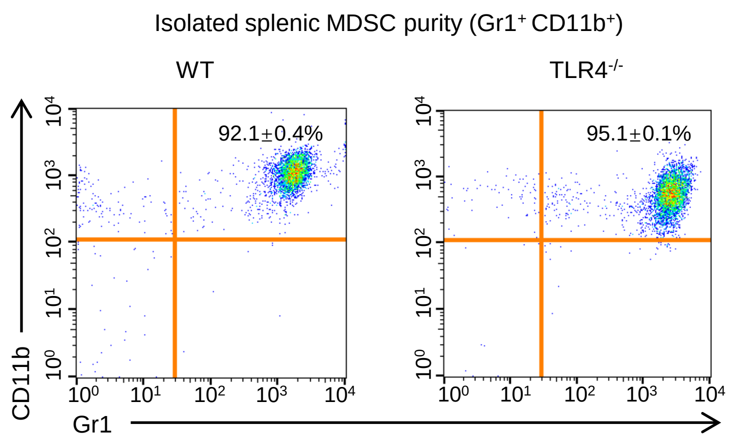

B

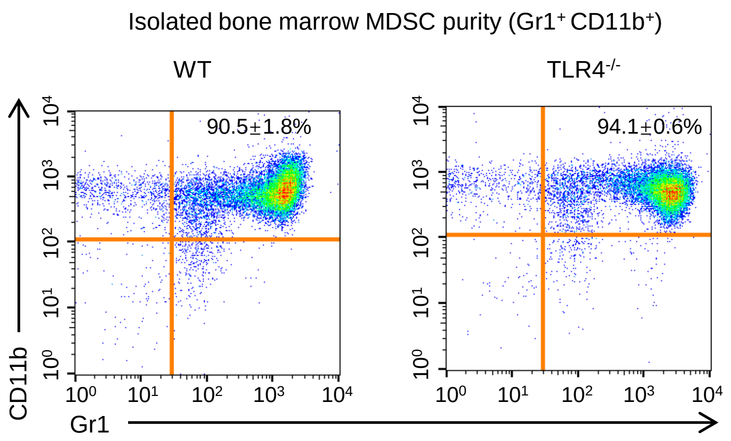

Supplementary Fig. 3

A

TLR4$^{-/-}$ *vs.* WT　　　TLR4$^{-/-}$ +CXCL10(300ng/ml) *vs.* TLR4$^{-/-}$

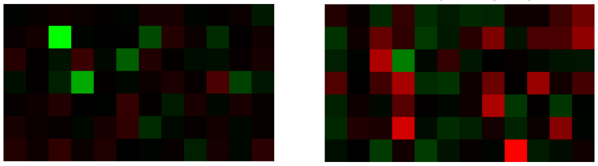

B

Fold Up- or Down-Regulation between compared groups of mouse bone marrow cells.

Well	Gene Symbol	Fold Up- or Down- Regulation			
		(WT+CXCL10)/WT	(TLR4$^{-/-}$ +CXCL10)/ (WT+CXCL10)	TLR4$^{-/-}$/ WT	(TLR4$^{-/-}$ +CXCL10)/ TLR4$^{-/-}$
A01	Myl12a	-1.3	1.38	-1.05	1.11
A02	Actn1	-1.63	1.84	1.1	1.02
A03	Actn3	-1.94	2	1.17	-1.13
A04	Actn4	-1.24	1.6	1.1	1.17
A05	Actr2	-1.19	1.09	1.04	-1.14
A06	Actr3	-1.2	1.05	-1.05	-1.08
A07	Akt1	-1.32	1.46	1.26	-1.13
A08	Arf6	-1.27	1.47	1.3	-1.12
A09	Arhgdia	-1.08	1.07	-1.05	1.05
A10	Arhgef7	-1.32	1.44	1.05	1.04
A11	Baiap2	-1.52	2.02	1.1	1.2
A12	Bcar1	-2.02	2.19	-1.3	1.41
B01	Capn1	-1.6	1.5	1.09	-1.17
B02	Capn2	-1.36	1.73	1.21	1.05
B03	Cav1	-2.03	-5.59	-15.49	1.36
B04	Cdc42	-1.22	1.34	-1.05	1.15
B05	Cfl1	-1.47	1.24	1	-1.19
B06	Crk	-1.26	1.21	1.03	-1.07
B07	Csf1	-1.44	-1.38	-2.23	1.12
B08	Cttn	-1.56	3.38	1.47	1.47
B09	Diap1	-1.38	1.45	1.1	-1.05
B10	Dpp4	-2.27	1.82	-1.56	1.26
B11	Egf	-2.25	2.86	1.01	1.26
B12	Egfr	-2.81	5.26	1.19	1.57
C01	Enah	-1.53	2.09	1.5	-1.1
C02	Ezr	-1.33	1.37	1.03	-1
C03	Fap	1.38	1.02	-1.22	1.72
C04	Fgf2	-1.21	1.5	1.82	-1.47
C05	Hgf	-1.69	1.51	-1.09	-1.03
C06	Igf1	1.02	-2.38	-2.72	1.17
C07	Igf1r	-1.58	2.08	1.4	-1.07
C08	Ilk	-1.16	1.21	1.04	-1
C09	Itga4	1.41	-1.58	-1.15	1.02
C10	Itgb1	1.03	-1.24	-1.17	-1.03
C11	Itgb2	-1.15	1.19	1.1	-1.06
C12	Itgb3	-1.01	1.2	1.27	-1.06
D01	Limk1	-1.66	1.82	-1.17	1.28
D02	Mapk1	-1.15	1.18	1.03	-1
D03	Met	1.19	-1.37	-1.39	1.21
D04	Mmp14	4.53	-16.62	-6.18	1.68
D05	Mmp2	1.33	-1.44	1.05	-1.14
D06	Mmp9	-1.21	-1.04	-1.08	-1.17
D07	Msn	-1.05	1.21	1.11	1.04
D08	Myh10	-2.07	3.83	1.33	1.39
D09	Myh9	-1.41	1.53	1.09	-1
D10	Mylk	-1.24	4.4	2.2	1.61
D11	Pak1	-1.59	-1.2	-2.01	1.05
D12	Pak4	-1.63	1.58	-1.27	1.23
E01	Pfn1	-1.3	1.26	1.07	-1.1
E02	Pik3ca	-1.21	1.45	1.15	1.04
E03	Plaur	-1.68	2.3	1.4	-1.02
E04	Plcg1	-1.19	1.58	1.01	1.3
E05	Pld1	-1.17	1.14	-1.03	1
E06	Prkca	-1.75	2.92	1.69	-1.02
E07	Pten	-1.49	1.63	1.05	1.04
E08	Ptk2	-1.69	2.2	-1.32	1.71
E09	Ptk2b	-1.22	1.34	1.3	-1.19
E10	Ptpn1	-1.02	-1.05	-1.08	1
E11	Pxn	-1.25	1.38	1.3	-1.17
E12	Rac1	-1.2	1.31	1.1	-1.01
F01	Rac2	-1.27	1.41	1.26	-1.13
F02	Rasa1	-1.23	1.51	1.12	1.1
F03	Rdx	-1.18	1.37	1.07	1.08
F04	Rho	-1.69	3.04	-1.08	1.93
F05	Rhoa	-1.14	1.43	1.3	-1.04
F06	Rhob	-1.29	1.87	1.65	-1.14
F07	Rhoc	-1.5	-1.14	-1.56	-1.1
F08	Rnd3	-1.31	1.28	-1.08	1.06
F09	Rock1	-1.39	1.34	1.07	-1.11
F10	Sh3pxd2a	-1.46	1.81	1.2	1.03
F11	Src	1.35	1.05	-1.07	1.52
F12	Stat3	-1.04	1.12	1.09	-1.01
G01	Svil	-1.34	1.83	1.43	-1.05
G02	Tgfb1	-1.34	1.55	1.16	-1
G03	Timp2	-1.54	2.1	1.64	-1.2
G04	Tln1	-1.31	1.54	1.11	1.06
G05	Vasp	-1.28	1.46	1.37	-1.21
G06	Vcl	-1.33	1.25	-1.01	-1.06
G07	Vegfa	-1.57	1.55	-1.03	1.02
G08	Vim	-1.19	1.29	1.09	-1.01
G09	Wasf1	-1.27	2.01	-1.38	2.2
G10	Wasf2	-1.25	1.46	1.27	-1.08
G11	Wasl	-1.34	1.22	-1.06	-1.04
G12	Wipf1	-1.15	1.88	1.55	1.05
H01	Actb	-1.31	1.39	1.04	1.02
H02	B2m	1.61	-2.16	-1.28	-1.05
H03	Gapdh	-1.07	1.05	1.06	-1.07
H04	Gusb	-1.03	1.09	1.08	-1.02
H05	Hsp90ab1	-1.11	1.35	1.08	1.13

Positive value and negative value represents up-regulation and down-regulation.

Supplementary Fig. 4

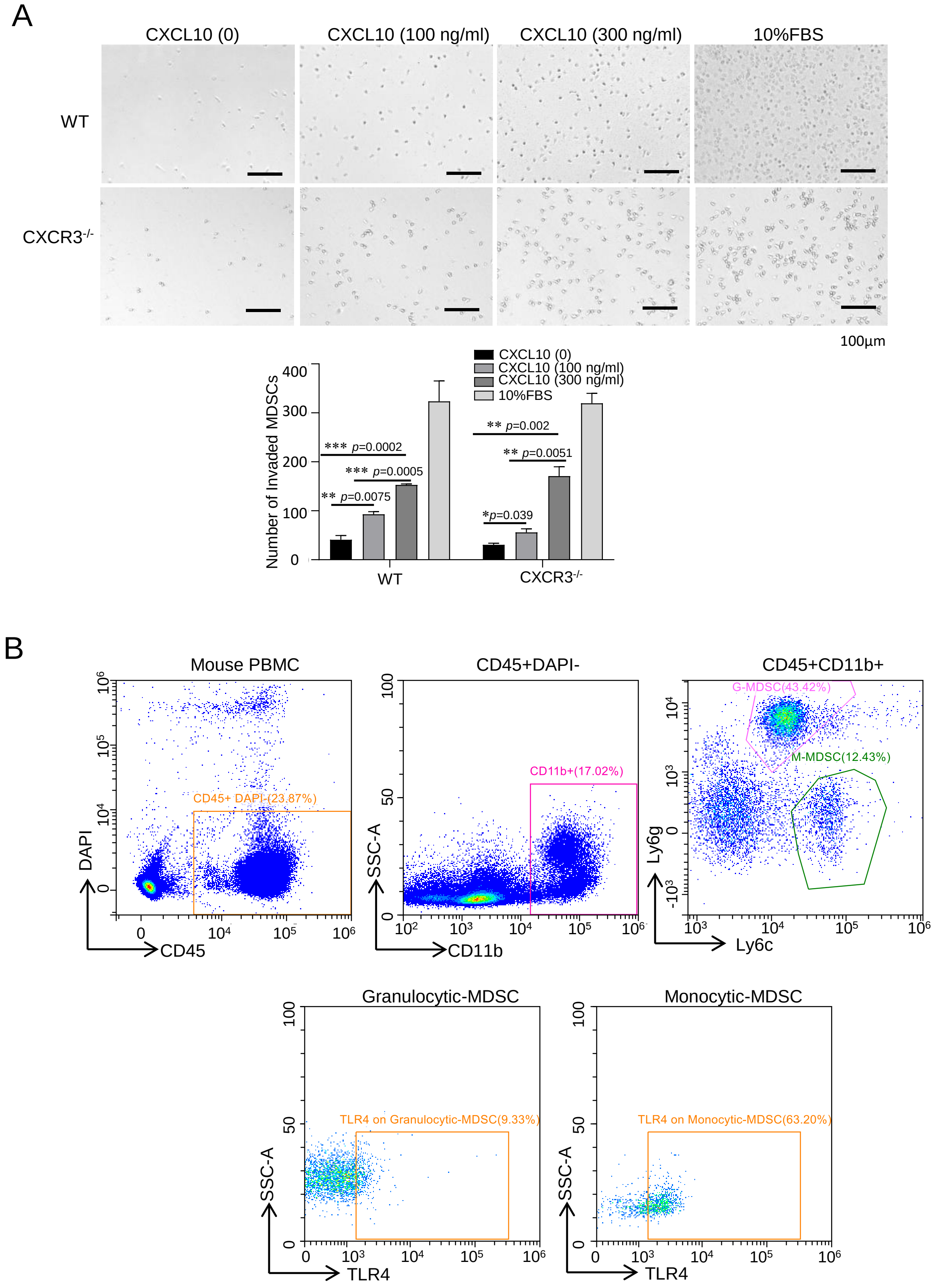

Supplementary Fig. 5

A

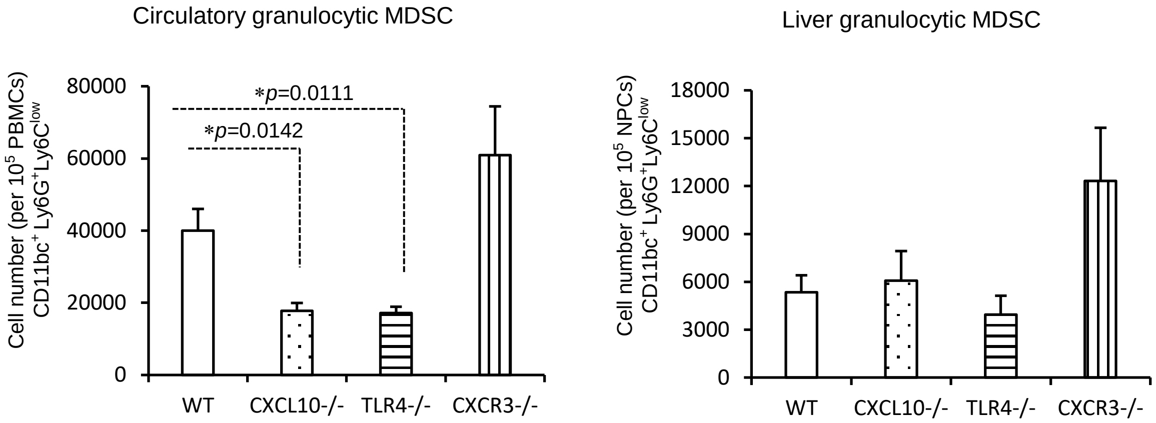

B

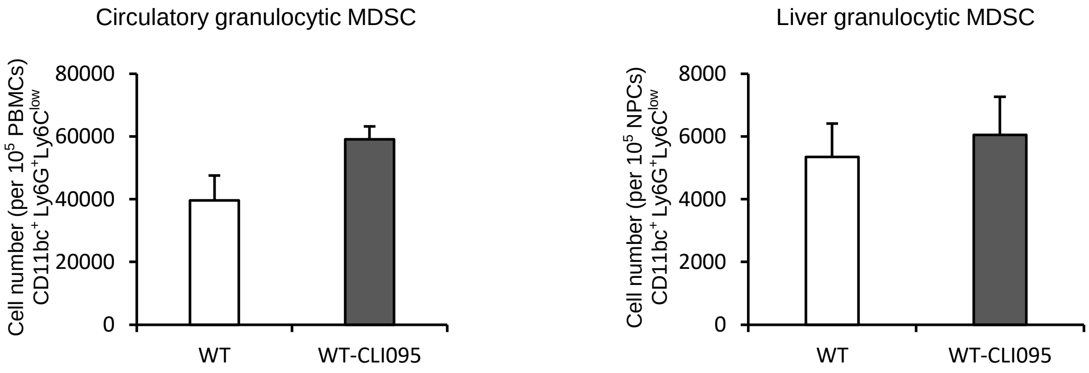

Supplementary materials and methods

Flow cytometry analysis

Flow cytometry was performed using a modified version of the previous protocol[26]. Human MDSCs were determined by CD33, CD13 (BD Pharmingen) and CD34 (Militenyi Biotech, Bergisch Gladbach, Germany). Rat MDSC population was detected by incubation with His48, CD80 (BD Pharmingen), CD11b/c (Invitrogen, CA, USA) antibodies. The mobilization of mouse $TLR4^+$ monocytic/granulocytic MDSCs was investigated by multicolor analysis of CD11b (BD Pharmingen), TLR4, Ly6G and Ly6C (Biolegend, CA, USA). Labeled cells were analyzed by BD FACS Calibur (BD Biosciences) and FlowJo (Treestar, San Carlos, CA, USA).

H&E and immunostaining

The detailed procedure of H&E, immunohistochemistry and immunofluorescent staining was applied using the previous methods with modification[26, 32, 33]. Antibodies against human CD33 (Leica, Milton Keynes, UK) and TLR4 (Abcam, MA, USA), rat CD11b/c (BD Pharmingen) and CD31 (Invitrogen), mouse CD31 and MMP14 (Invitrogen) were used. The Hamamatsu imaging system (Hamamatsu Photonics, Shizuoka, Japan) and Carl Zeiss LSM 780 imaging System (Carl Zeiss, Jena, Germany) was used to count the positive cells and acquire the images.

Quantitative Real-Time Polymerase Chain Reaction (qRT-PCR)

Total RNA was extracted from human liver specimens and rat liver tissues using Trizol Reagent (Invitrogen). Complementary DNA was synthesized from 1μg total RNA using High Capacity cDNA Reverse Transcription Kits (Applied Biosystems, CA, USA). The qRT-PCR was carried out using an Applied Biosystems 7900HT Fast Real-time PCR system with primers listed in

Supplement Table 1 and Fast Start SYBR Green Master Mix (Roche Diagnostic Systems, NJ, USA).

MDSC transwell assay

Freshly isolated splenic MDSCs ($1x10^5$) supplemented with 10ng/mL granulocyte-macrophage colony-stimulating factor and 10 ng/mL interleukin-4 in RPMI1640 were seeded on the upper chambers of 24-well Transwell inserts (5 µm polycarbonate membrane, Costar, Corning, NY, USA). In the lower chambers, 100 ng/ml, 300 ng/ml CXCL10 recombinant protein (R&D Systems, Minneapolis, MN, USA) and 10%FBS were added. MMP14 antibody (30 µg/ml, EMD Millipore, CA, USA) was incubated with wild type MDSCs for 30 mins before seeding to the chambers. The migrated MDSCs found in the lower chambers were observed and counted after 16 hours of stimulation by a microscope (EVOS FL/Life technologies, NY, USA). Experiments were performed in triplicate.

RT2 profiler PCR array

Bone marrow isolated MDSCs from wild type and *TLR4*$^{-/-}$ mice were cultured with/without CXCL10 (300 ng/ml) recombinant protein for 16 hours. Total RNA was extracted from freshly collected bone marrow MDSCs (Promega, WI, USA). The complementary DNA was synthesized from 250 ng total RNA with RT2 first strand Kit (Qiagen, CA, USA). The RT2 profiler PCR array was performed by Applied Biosystem Viia7 Fast real time PCR system using RT2 SYBR Green ROX FAST Mastermix (Qiagen) according to the manufacturer's protocols. The array results were analyzed by Qiagen analysis system.

Supplementary figure legends

Supplementary Fig. 1 No significant change of granulocytic MDSCs by the deficiency of CXCL10, TLR4 or CXCR3 in mouse IRH model. A No significant alteration of circulatory and liver granulocytic MDSCs in *CXCL10*$^{-/-}$ mice compared to wild type mice. **B** No noticeable change of mobilization and recruitment from granulocytic MDSC in *TLR4*$^{-/-}$ mice. **C** No significant change of circulatory and hepatic granulocytic MDSCs by knockout of CXCR3. N=5/group; Error bars indicate Standard Error of Mean; MDSC, myeloid-derived suppressor cell; IRH, ischemia/reperfusion plus major hepatectomy; WT, wild type.

Supplementary Fig. 2 The purity of primary MDSCs isolated from mice by flow cytometry. A The purity of splenic MDSCs freshly isolated from wild type and *TLR4*$^{-/-}$ mice. **B** The purity of MDSCs freshly isolated from the bone marrow of wild type and *TLR4*$^{-/-}$ mice. N=5; MDSC, myeloid-derived suppressor cell; WT, wild type.

Supplementary Fig. 3 The fold change of MDSC motility genes from wild type and *TLR4*$^{-/-}$ mice by RT2 screening. A The comparisons of mouse MDSC motility genes by *TLR4*$^{-/-}$ *vs.* WT, *TLR4*$^{-/-}$+CXCL10 (300 ng/ml) *vs.* *TLR4*$^{-/-}$. **B** The fold changes of MDSC motility genes by comparison of WT+CXCL10(300 ng/ml) *vs.* WT, *TLR4*$^{-/-}$+CXCL10 (300 ng/ml) *vs.* WT+CXCL10 (300 ng/ml), *TLR4*$^{-/-}$ *vs.* WT and *TLR4*$^{-/-}$ +CXCL10 (300 ng/ml) *vs.* *TLR4*$^{-/-}$. MDSC, myeloid-derived suppressor cell; WT, wild type.

Supplementary Fig. 4 No significant change of CXCR3$^{-/-}$ MDSC migration induced by CXCL10 compared with wild type ones and higher expression of TLR4 on monocytic MDSCs. A More CXCR3$^{-/-}$ MDSCs transferred to the bottom well in a CXCL10 dosage-dependent manner, similar with the wild type ones. N=3. **B** Dot plots of TLR4 levels on monocytic and

granulocytic MDSCs in mouse IRH model. Error bars indicate Standard Error of Mean; *$p<0.05$, **$p<0.01$, ***$p<0.001$. MDSC, myeloid-derived suppressor cell; IRH, ischemia/reperfusion plus major hepatectomy; WT, wild type.

Supplementary Fig. 5 The alteration of granulocytic MDSCs by the deficiency of CXCL10, TLR4, CXCR3 and inhibition of TLR4 in mouse IRH with tumor recurrence model. **A** No apparent change of liver granulocytic MDSCs although there is a significant decrease of circulatory granulocytic MDSCs in *CXCL10*$^{-/-}$ or *TLR4*$^{-/-}$ mice compared to wild type ones. **B** No obvious alteration of granulocytic MDSC mobilization and recruitment by TLR4 inhibition in mouse IRH with tumor recurrence model. N=5/group; MDSC, myeloid-derived suppressor cell; IRH, ischemia/reperfusion plus major hepatectomy; WT, wild type.

Supplementary tables

Supplementary Table 1 Primers used for the amplification of genes.

**Primers**	**Forward sequence (5' to 3')**	**Reverse sequence (5' to 3')**
Human CXCL10	GAATCGAAGGCCATCAAGAA	CCTCTGTGTGGTCCATCCTT
Human TLR4	ATTTATCCAGGTGTGAAATCCAG	ATTATTAAGGTAGAGAGGTGGCTTAGG
Rat CXCL10	AAGCGGTGAGCCAAAGAAGGTCAA	AGCCGCACACTGGGTAAAGGGA
Rat TLR4	ACCGCTGGGAGAGAAGGGGC	CCCTTGCTCCTCCTGGAGTCACA
Rat CXCR3	TCACGGCAAGTTCCCAACCACA	AGCACTTGACGTTCACTGACCTCA

Supplementary Table 2 Antibodies used for the staining.

Name	Citation (PMID)	Supplier	Cat no.	Clone no.
Anti-CD33	8822961	BD Pharmingen	555450	WM53
Anti-CD13	2665173	BD Pharmingen	557454	WM15
Anti-CD34	26395069	Militenyi Biotech	130-081-001	AC136
Anti-His48	2046327	BD Pharmingen	554907	HIS48
Anti-CD80	9237108	BD Pharmingen	555012	3H5
Anti-CD11b/c	-	Invitrogen	MA5-17507	OX-42
Anti-CD11b	6184305	BD Pharmingen	557397	M1/70
Anti-Gr1	-	Stem Cell	60028FI.1	RB6-8C5
Anti-TLR4	16946018	Biolegend	145408	SA15-21
Anti-Ly6G	23696668	Biolegend	127605	1A8
Anti-Ly6C	26519532	Biolegend	128010	HK1.4
Anti-CD33	23708142	Leica	NCL-L-CD33	PWS44
Anti-TLR4	28098138	Abcam	ab22048	76B357.1
Anti-CD11b/c	1672643	BD Pharmingen	550299	OX-42
Anti-CD31	25079331	Invitrogen	PA5-16301	-
Anti-CD31	28707321	Invitrogen	MA1-40074	-
Anti-MMP14	19020757	Invitrogen	PA5-13183	-
Anti-MMP14	26091717	EMD Millipore	MAB3328	LEM-2/15.8
